# Deciphering the Enigmatic Function of *Pseudomonas* Metallothioneins

**DOI:** 10.3389/fmicb.2020.01709

**Published:** 2020-07-22

**Authors:** Jelena Habjanič, Anugraha Mathew, Leo Eberl, Eva Freisinger

**Affiliations:** ^1^Department of Chemistry, University of Zurich, Zurich, Switzerland; ^2^Department of Plant and Microbial Biology, University of Zurich, Zurich, Switzerland

**Keywords:** metallothionein, *Pseudomonas fluorescens*, stationary phase, long-term viability, zinc, cadmium

## Abstract

Metallothioneins (MTs) are low molecular weight, Cys-rich proteins that sequester both essential and non-essential metal ions. Despite being highly conserved in the *Pseudomonas* genus of Gram-negative bacteria, knowledge of their physiological function in this species is scarce. Using the strain *P. fluorescens* Q2-87 as a model organism, we investigated the role of a conserved MT in zinc homeostasis, cadmium detoxification as well as its implications in stress response. We show that MT expression is only induced in the stationary phase and provides a fitness benefit for long-term starvation survival, while it is not required for metal resistance and acquisition, oxidative or nitrosative stress response, biofilm formation or motility.

## Introduction

Zn^II^ is considered to be an essential metal ion playing a role as a catalytic or structural cofactor of many key enzymes involved in DNA replication, protein synthesis, and other crucial enzymatic reactions in all organisms. However, zinc is toxic in excess and bacteria have evolved various mechanisms to tightly regulate the intracellular concentration of this metal, keeping the free zinc concentration extremely low (femto- to picomolar range) (Blindauer, 2015; Watly et al., 2016; Mikhaylina et al., 2018). Cd^II^ is highly toxic for most organisms and its acute toxicity is closely associated with oxidative stress (Maret and Moulis, 2013). In addition, while Cd^II^ is highly similar to Zn^II^ with respect to coordination preferences and redox inactivity, it is also more thiophilic and hence displaces Zn^II^ efficiently in the corresponding biologically relevant binding sites. Therefore, bacteria have developed various metal uptake and efflux systems to finely regulate metal ion homeostasis.

Metallothioneins (MTs), low molecular weight Cys-rich proteins, are one of the important partakers in maintaining zinc or copper homeostasis as well as cadmium detoxification in many living organisms (Blindauer, 2014). They have unique metal binding properties that ultimately result in the formation of characteristic metal-thiolate clusters. Due to the high abundance of Cys residues, they can also actively participate in scavenging reactive oxygen and nitrogen species (Ruttkay-Nedecky et al., 2013). The presence of MTs in bacteria (bacMTs) was considered as an exception more than a rule for a long time, but their ubiquity became evident in the last decade due to sequencing efforts of numerous bacterial strains (Blindauer, 2009). However, a biological function of only two bacMTs has been investigated to date. SmtA from the Gram-negative cyanobacterium *Synechococcus elongatus* plays an essential role in zinc detoxification (Olafson et al., 1988; Robinson et al., 1990), while a MT from the pathogenic Gram-positive bacterium *Mycobacterium tuberculosis* (MymT) is crucial for copper detoxification (Gold et al., 2008).

**FIGURE 1:**
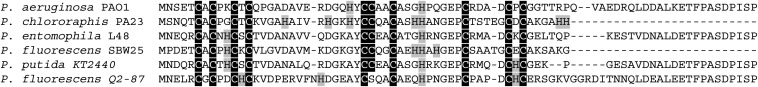
*Pseudomonas* MTs. Amino acid sequences alignment of MTs from different *Pseudomonas* species. Cys residues are highlighted in black, His residues in gray.

**FIGURE 2:**
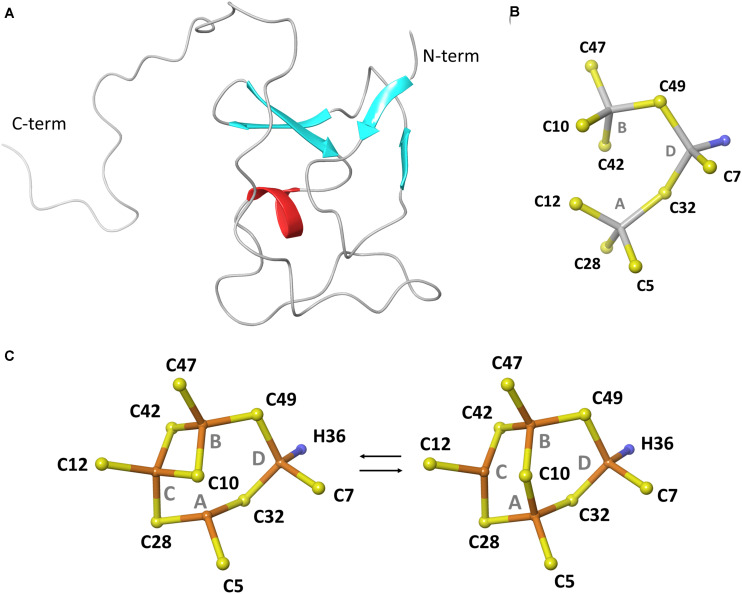
Structure of MT from *P. fluorescens* Q2-87. **(A)** Representative structure of the protein backbone fold. **(B)** Topology of Zn_3_Cys_9_His cluster. **(C)** Topology of Cd_4_Cys_9_His cluster. **(B,C)** are reproduced from Habjanic et al. (2018) with permission from The Royal Society of Chemistry.

Bioinformatic analysis of newly sequenced bacterial genomes has revealed that MTs are conserved in 90% of the *Pseudomonas* species (Winsor et al., 2016), including pathogenic (e.g., *P. aeruginosa* and *P. entomophila*) and non-pathogenic strains (e.g., *P. fluorescens* and *P. putida*), which implies their possible involvement in physiological processes necessary for survival in host and non-host environments (Figure 1). This emerging group of *Pseudomonas* MTs (PsdMTs) shows a conserved Cys distribution pattern with cyanobacterial MTs, suggesting similar metal preferences and biological functions. However, PsdMTs contain unique features both in their amino acid sequences (such as high abundance of His residues, an intrinsically disordered C-term tail, and variations of the coordinating residues) and on the genomic level (see below). Recently, we determined the first 3D structure of a PsdMT from the *P. fluorescens* strain Q2-87 (PflQ2 MT) (Habjanic et al., 2018). Despite of the interesting novel features mentioned above, the overall structure of PflQ2 MT is similar to the structure of SmtA from *S. elongatus* (Blindauer et al., 2001) and coordination of four cadmium ions is observed (Figure 2). The binding capacity for zinc ions is, however, reduced to three in PflQ2MT compared to four in SmtA.

An important difference between SmtA and *Pseudomonas* MTs is their genetic organization. A number of cyanobacterial MTs including SmtA have a defined operon structure consisting of the MT, a zinc-dependent divergently transcribed repressor (SmtB) and an operator-promoter region in the intergenic region (Huckle et al., 1993; Morby et al., 1993; Turner et al., 1996; Blindauer, 2008). In contrast, the *Pseudomonas* MT operon lacks metal-responsive regulators and its common promoter sequences. Furthermore, genes located upstream or downstream of the MT gene appear to be part of the same operon and code for proteins of unknown functions.

The genus *Pseudomonas* contains the largest number of described species among the Gram-negative bacteria. The members of the genus are metabolically versatile with the exceptional ability to survive in heterogeneous, dynamic and hostile environments (Silby et al., 2011; Gomila et al., 2015). The last decade saw an increased interest in understanding the heavy metal metabolism in *Pseudomonas*, and in particular the zinc homeostasis, owing to the importance of heavy metal homeostasis at the host/pathogen interface (Higham et al., 1984; D’Orazio et al., 2015; Pederick et al., 2015; Palmer and Skaar, 2016; Mastropasqua et al., 2017). It was found that the MT from *P. aeruginosa* PAO1 is present in biofilms (Hentzer et al., 2005; Waite et al., 2005, 2006) and its transcription was found to be upregulated in burn and chronic wounds (Turner et al., 2014). Similarly, the presence of elevated MT transcripts was confirmed in *P. aeruginosa* N6P6 biofilms and was also detected in response to elevated Pb^II^ concentrations (Kumari and Das, 2019). However, in all these reports, the specific functions of the respective MT or its relevance for survival were not further investigated. To unravel the biological function of *Pseudomonas* MTs and their regulation, we used the *P. fluorescens* strain Q2-87 as a model since the structure of PflQ2 MT was determined recently (Figure 2; Habjanic et al., 2018). In this study, we focused on the involvement of this PsdMT in maintaining zinc homeostasis, including zinc limitation and excess, and its potential role in cadmium detoxification and stress response.

## Materials and Methods

### Bacterial Strains, Plasmids, and Media

Bacterial strains and plasmids used in this study are listed in Supplementary Tables S1, S2, while primers are listed in Supplementary Table S3. The model strain used in this study, *P. fluorescens* Q2-87, was isolated from wheat rhizosphere (Washington, United States) (Loper et al., 2012). Genomic DNA was extracted using the DNeasy Blood & Tissue Kit (Qiagen, Hilden, Germany) while plasmid DNA was extracted with the QIAprep Spin Miniprep kit (Qiagen) using standard protocols. All solutions and media were prepared with distilled H_2_O and sterilized for 20 min at 121°C and 1.2 bar unless stated otherwise. For DNA work and solutions that cannot be autoclaved Milli-Q water was used, and solutions were filtered using a 0.22 μm vacuum filtration system (Steriflip, Merck Millipore, United States). For solid media, 1.5% (w/v) of agar was added prior to autoclaving. *P. fluorescens* strains were grown at 30°C in lysogeny broth (LB) medium or the ABG minimal media (Clark and Maale, 1967) that was modified by replacing the phosphate buffer component with 250 mM HEPES, pH 7.4 and supplemented additionally with 0.2% of yeast extract solution in order to boost the growth. Both LB and modified ABG minimal media contain similar amounts of Zn^II^ (13 and 10 μM, respectively).

*E. coli* strains SY327 λpir and Top 10 were used as cloning hosts and were grown at 37°C in LB. Antibiotics were added to the following final concentrations: 50 μg mL^–1^ trimethoprim, 10 μg mL^–1^ tetracycline, 20 μg mL^–1^ gentamycin, 50 μg mL^–1^ kanamycin, 100 μg mL^–1^ ampicillin. Bacterial cultures were stored in 25% glycerol at −80°C.

The metal ion stocks of cadmium and zinc ions were prepared by dissolving ZnCl_2_ and CdCl_2_⋅2.5 H_2_O (Sigma Aldrich, Buchs, Switzerland) in Milli-Q water. The final concentration of metal ion stock solutions was measured using a flame atomic absorption spectrometer (F-AAS; AA240FS spectrometer (Agilent Technologies AG, Basel, Switzerland).

### Construction of MT Gene Knockout

In order to ensure targeted, non-polar unmarked gene deletion, a mutagenesis system based on the homing endonuclease I-SceI was used (Supplementary Figure S1; Flannagan et al., 2008). Since this mutagenesis system was to the best of our knowledge for the first time applied on a *P. fluorescens* strain, conditions and the antibiotic selection procedure had to be thoroughly optimized. Since *P. fluorescens* is trimethoprim resistant (Tp^R^), additionally a tetracycline antibiotic cassette was cloned into the suicide plasmid pGPI-SceI that has an I-SceI recognition site. The tetracycline resistance cassette was amplified from the shuttle plasmid pMe6032 (Heeb et al., 2002) using primers listed in Supplementary Table S3 and inserted into the pGPI-SceI plasmid using the Pst1 restriction site. Clones were selected using tetracycline, and the product (pGPI-SceI_tetR) was additionally confirmed by polymerase chain reaction (PCR) using the same primers used for the amplification of the tetracycline resistance cassette.

Regions of homology, MT_up and MT_down, that flank the MT gene targeted for the deletion were amplified using primers listed in Supplementary Table S3 and cloned into a newly constructed pGPI-SceI_tetR plasmid. Clones were selected using tetracycline and additionally confirmed by PCR using the same primers used for the amplification of homology regions. The constructed plasmid was introduced by triparental mating into *P. fluorescens* Q2-87 using the pRK2013 helper plasmid, which resulted in the integration of the plasmid into the genome by homologous recombination. The product of the first conjugation step was selected using tetracycline and confirmed by PCR using two sets of *Pseudomonas* specific primers, i.e., MT_up_Kpn1_fwd and MT_dn_EcoR1_rev (Supplementary Table S3).

The plasmid pDAI-SceI, which expresses the I-SceI nuclease and carries a gentamycin resistance (Gm^R^), was inserted into the product of the first step by triparental mating using pRK2013 as a helper strain. I-SceI expression resulted in cleavage at the I-SceI recognition site, producing a double strand break in the genomic DNA. This DNA break stimulates the host recombination/repair machinery resulting in two possible scenarios. The first scenario is removing the intervening sequences between MT_up and MT_dn resulting in the MT gene deletion, while the second scenario includes recombination that occurs between the MT_up and MT_dn region resulting in repair and recovery of the wild type form of *P. fluorescens* Q2-87. The final product, ΔMT *P. fluorescens* Q2-87, was confirmed with PCR (Supplementary Figure S2) and by sequencing.

### Construction of Transcriptional lacZ Fusions and β-Galactosidase Assay

The BPROM prediction server for bacterial promoters (Solovyev and Salamov, 2011) predicted two possible promoters (Supplementary Figure S3) forming two probable promoter regions, a bigger (L) and a smaller (S) one. Both regions were amplified using primers listed in Supplementary Table S3, digested with *Xho*I and *Hin*dIII restriction enzymes, and inserted into the reporter plasmid pSU11 (Malott et al., 2009). The resulting plasmids (pSU11/Lprom-lacZ; pSU11/Sprom-lacZ) were transferred to *P. fluorescens* Q2-87 by triparental mating using the helper plasmid (pRK2013). The resulting transconjugants were verified with two sets of primers, the forward and reverse primers used for amplification of promoter regions, and with a set including the forward primer from the amplification and reverse primer specific for the pSU11 plasmid (Supplementary Table S3).

The β-galactosidase assay was performed as previously described with minor modifications (Miller and Mekalanos, 1988). Briefly, cells were harvested and resuspended in Z-buffer (see appendix, 1 mL), and the OD_600_ was measured. Cells were lysed using chloroform (25 μL), sodium dodecyl sulfate (SDS; 25 μL, 0.01%) and briefly vortexed. The resulting mixture was incubated for 10 min at 28°C. The reaction was initiated by adding ortho-Nitrophenyl-β-galactosidase (ONPG; 200 μL, 10 mg/mL), and stopped with Na_2_CO_3_ (500 μL, 1 M). Cell debris was removed by centrifugation (10 min, 15,000 rpm) and the absorbance at 420 nm was measured. The β-galactosidase activity was calculated using the following equation:

$$\mathrm{Activity}\,\left[\mathrm{Miller}\,\mathrm{units}\right]=\frac{1000\times {\mathrm{OD}}_{420}}{\mathrm{time}\,\left[\min\right]\times V\,\left[\mathrm{mL}\right]\times 0\,D_{600}}$$

### Growth Assay and Determination of MIC

The minimum inhibitory concentration (MIC) of *P. fluorescens* Q2-87 and its ΔMT mutant was determined by measuring the OD_600_ after 12 h incubation at 30°C, 220 rpm upon addition of different concentrations of Zn^II^ and Cd^II^ ions. Additionally, the growth of both strains was monitored in 1 h increments for a total of 12 h by measuring the OD_600_ in the presence of 25 μM Cd^II^ or 50 μM Zn^II^. In both experiments, cultures without the addition of metal ions were used as a control.

### Biofilm Formation and Quantification

An overnight bacterial culture was subcultured into modified ABG-media to a starting OD_600_ of 0.03 and transferred in 200 μL aliquots into a round-bottom 96-well microtiter plate (Sarstedt, Switzerland). The plate was incubated for 48 h at 10°C. After incubation, growth was checked by measuring OD_550_ in the plate reader (Biotek, PowerWave XS2), 100 μL of 0.1% (w/v) crystal violet (triarylmethane) solution was added into each well and incubated for 30 min at room temperature. Afterward, the media was discarded, wells washed carefully with distilled water in order to remove the planktonic cells and the plate was left at room temperature to dry. To solubilize the crystal violet, 200 μL of dimethyl sulfoxide (DMSO) was added to each well and incubated for 20 min at room temperature. The content of each well was transferred into a flat-bottom 96-well plate and the absorbance at 570 nm was measured in order to quantify the biofilms.

### Motility Assay

Swarming and swimming motility was determined on agar plates using modified ABG-media using 0.4% (w/v) and 0.3% (w/v) of agar respectively (Eberl et al., 1996). Plates were inoculated with overnight bacterial culture by stabbing with a sterile toothpick for swimming motility or spotting 2.5 μL of bacterial culture in the center for swarming motility. The plates were incubated at 28°C for 24 h.

### Tagging Bacteria With Mini-Tn7 System

*P. fluorescens* Q2-87 as well as the ΔMT knock strain were tagged with mCherry/Gn^R^ using the mini-Tn7 system. For tri-parental mating, recipient (*P. fluorescens* Q2-87/ΔMT), donor [*E. coli* S17-1 containing mini-Tn7 plasmid (pMRE100)] and helper (*E. coli* S-17-1 carrying pUX-BF13 plasmid) strains were used. The products of the conjugation were selected using both ampicillin and gentamycin.

### Bacterial Competition Assay

*P. fluorescens* strains (Q2-87 ΔMT, Q2-87_mCherry) were grown overnight at 30°C in modified ABG media (see above). Each culture for the competition assay was added to modified ABG media supplemented with 50 μM Zn^II^ to a final OD_600_ of 0.02. After a growth period of 24–120 h at 30°C, serial dilutions (10^–1^ to 10^–7^) of bacterial cultures were plated on LB agar plates (with and without gentamycin). Plates were incubated overnight at 30°C, and the number of viable colony-forming units (CFU) was counted and used to calculate the CFU/mL for all culture tested. All experiments were carried out at least three times.

## Results and Discussion

### PflQ2 MT Is Dispensable for Zinc Homeostasis and Cadmium Detoxification

**FIGURE 3:**
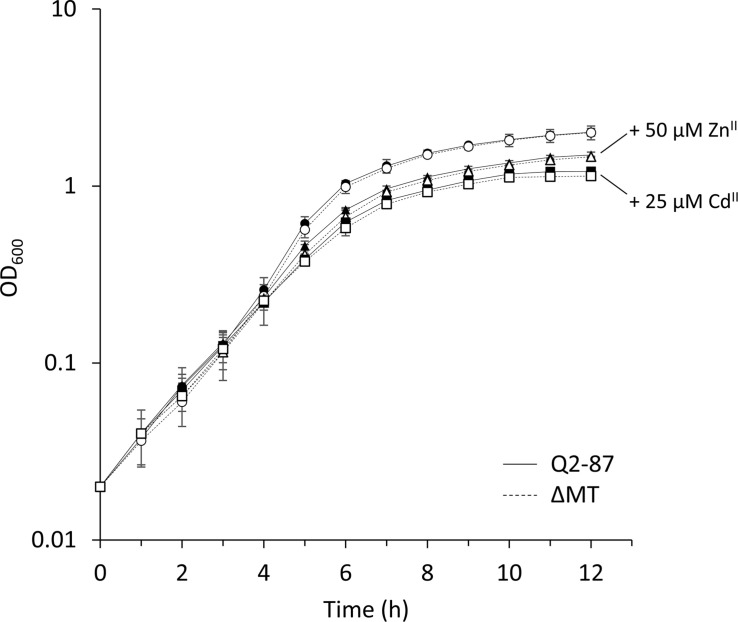
The effect of Zn^II^ and Cd^II^ on *P. fluorescens* growth. Growth of *P. fluorescens* Q2-87 and the ΔMT mutant was compared by optical density measurements at 600 nm upon addition of 50 μM Zn^II^ or 25 μM Cd^II^. Data are presented as the mean of three independent experiments and the standard deviation is given as error bars. See Supplementary Figure S5 for linear growth scale.

To directly assess the role of the putative metallothionein PflQ2 MT of *P. fluorescens* Q2-87, a mutant strain defective in the production of this MT (ΔMT) was constructed. Since previous *in vitro* studies suggested that PflQ2 MT binds zinc and cadmium ions (Habjanic et al., 2018), the role of PflQ2 MT in metal resistance was assessed by determining the minimal inhibitory concentrations (MIC) of the wild type and the mutant strain to these two metal ions. Both the Q2-87 wild-type and the DMT mutant strains showed a growth impairment already at low Zn^II^ and Cd^II^ concentrations of 25 and 5 μM, respectively (Supplementary Figure S4). However, no significant difference in growth between the two strains was observed with or without the addition of inhibitory concentrations of Zn^II^ and Cd^II^ (Figure 3 and Supplementary Figure S5). Similar results were also obtained in the presence of different concentrations of the metal chelator EDTA (ethylenediaminetetraacetic acid) or TPEN [N, N,N’,N’-tetrakis(2-pyridinylmethyl)-1,2-ethanediamine] (data not shown).

The similar growth pattern of the wild type and mutant strain under Zn^II^/Cd^II^ excess as well as metal-limited conditions suggest that PflQ2 MT is neither critical for metal resistance nor for metal uptake in *P. fluorescens* Q2-87.

### MT Expression Is Elevated in the Stationary Growth Phase

To elucidate the putative function of PflQ2 MT in growth and survival of *P. fluorescens* under different conditions, we analyzed the activity of the PflQ2 MT promoter. Two putative bacterial sigma70-dependent promoter regions were identified using BROM (Solovyev and Salamov, 2011). One of these putative promoters was found to be located 922 nucleotides upstream of the MT gene (Lprom), while the second one was identified 594 nucleotides upstream of the MT gene (Sprom) (Supplementary Figure S3). To study the regulation of MT gene expression, transcriptional fusions of the two putative promoter regions to the reporter gene *lacZ* were generated. The resulting fusions and the empty vector as a control were transformed into the *P. fluorescens* wild-type strain and β-galactosidase activities were determined under various growth conditions, including the addition of different d-block metal ions (Zn^II^, Cd^II^, Cu^II^, Hg^II^), oxidative stress induced by addition of hydrogen peroxide or tert-butyl hydroperoxide, and nitrosative stress induced by peroxynitrite and S-nitrosoglutathione. The results show that Sprom_pSU11 and the vector control display very low β-galactosidase activities, while Lprom_pSU11 displays significantly higher activities depending on the respective concentrations and the growth conditions (Supplementary Figure S6). Therefore, Lprom is the most probable MT promoter candidate, and the respective transcriptional fusion was used in all subsequent experiments.

**FIGURE 4:**
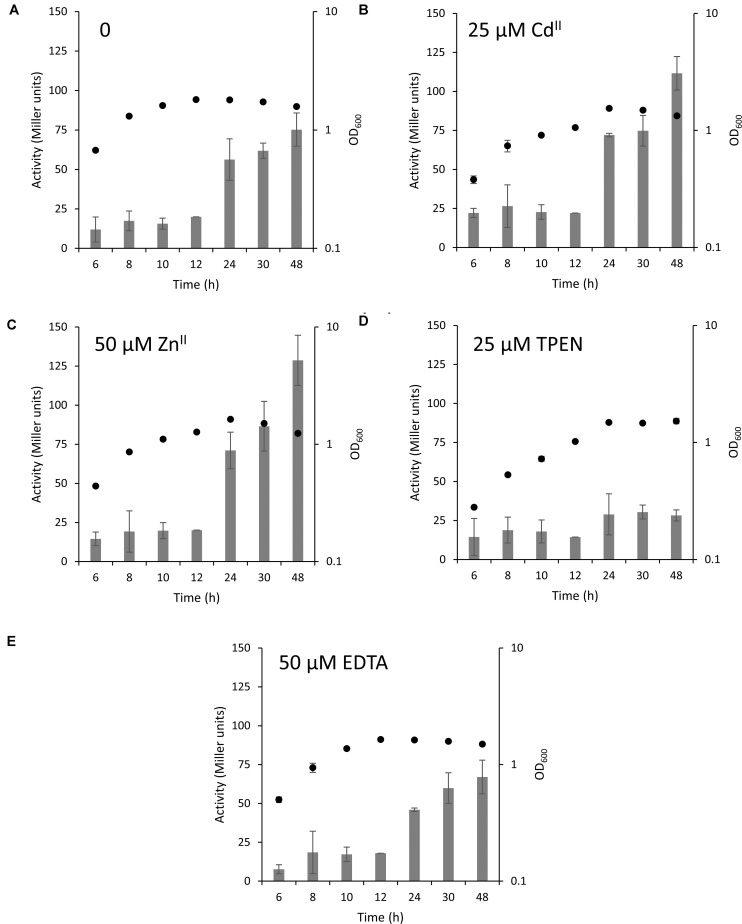
MT expression in different stages of bacterial growth. MT expression in the wild type Q2-87 in modified ABG medium is depicted in form of bars in Miller units with **(A)** no addition of metals, **(B)** 25 μM Cd^II^, **(C)** 50 μM Zn^II^, **(D)** 25 μM TPEN or **(E)** 50 μM EDTA. The secondary y-axis on the right shows the OD_600_ values of bacterial culture at the time samples were taken (black full circles). See Supplementary Figure S7 for linear growth scale.

We next grew this strain in the absence or presence of inhibitory concentrations of Zn^II^ (50 μM), Cd^II^ (25 μM), TPEN (25 μM), and EDTA (50 μM) for 48 h (Figure 4 and Supplementary Figure S7). Surprisingly, the MT promoter activity was only slightly increased in the exponential phase of growth, while it increased 10 to 20-fold when the bacteria entered the stationary phase after 24 to 48 h. However, this difference was not observed in media supplemented with TPEN where the level of MT expression remained the same throughout the experiment. While the stability constants of EDTA and TPEN for Zn^II^ are in the same range (10^15.4^ and 10^15.2^ M^–1^, respectively) (Radford and Lippard, 2013), TPEN readily permeates cell membranes (Arslan et al., 1985) and accordingly limits the intracellular availability of Zn^II^.

**FIGURE 5:**
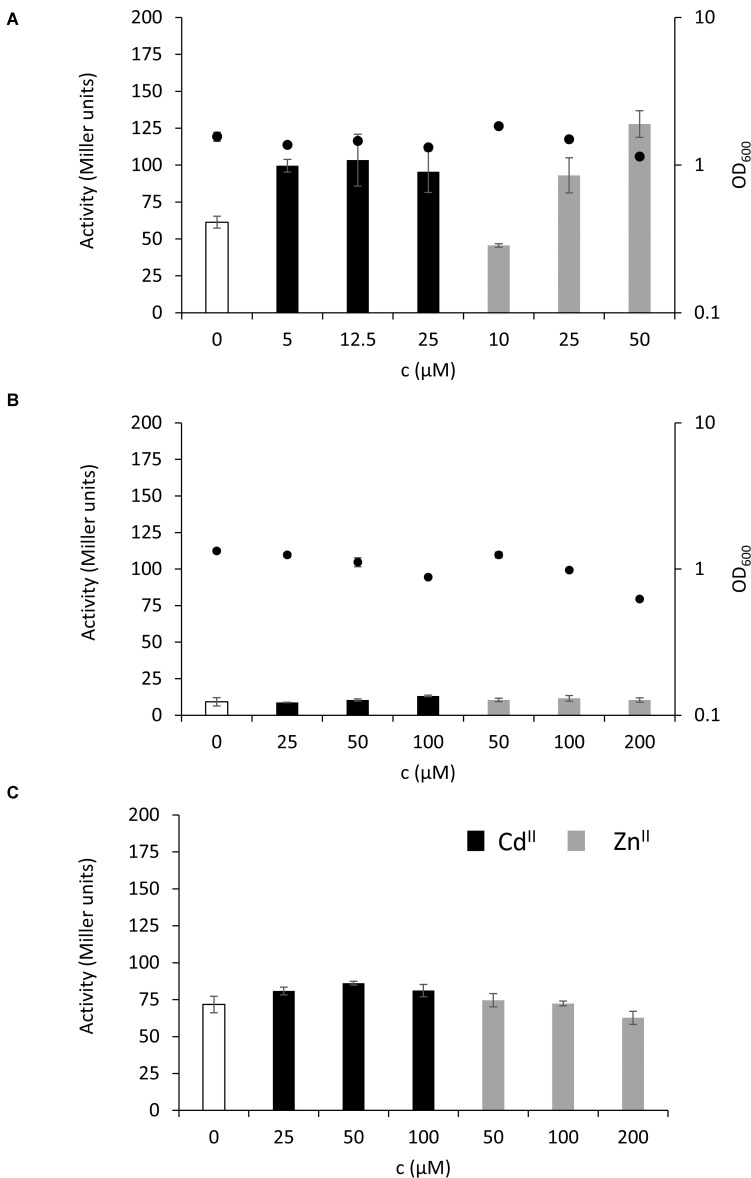
Level of MT expression in the stationary and exponential phase of growth. MT expression without additional metal ions (white bars) or in the presence of different concentrations of Zn^II^ (gray bars) or Cd^II^ (black bars) during mid-exponential phase of bacterial growth is given in Miller units and depicted after **(A)** 48 h incubation or **(B)** 2 h incubation. In **(C)** the different concentrations of Zn^II^ or Cd^II^ were added after 48 h of bacterial growth followed by 2 h incubation. The secondary y-axis on the right in **(A,B)** represents OD_600_ values at the time samples were taken (black full circles). See Supplementary Figure S8 for linear growth scale.

The key finding that the MT is highly expressed in the stationary phase of *P. fluorescens* growth under normal conditions and in the presence of inhibitory concentrations of Cd^II^ and Zn^II^ suggests that PflQ2 MT is probably important for long-term survival of the bacterium. To further evaluate this hypothetical role, we tested if increasing cadmium and zinc concentrations result in even higher MT promoter activity after 48 h of incubation. No correlation between the promoter activities and Cd^II^ concentrations was observed, while MT expression further increased with higher concentrations of Zn^II^ concomitant with a slight reduction of growth (Figure 5A and Supplementary Figure S8A). Although the addition of inhibitory concentrations of Zn^II^ and Cd^II^ during the exponential phase (OD_600_ = 0.55) and incubation for 2 h did not induce MT transcription, a decline in growth with increasing metal concentrations was observed (Figure 5B and Supplementary Figure S8B). Likewise, addition of the same inhibitory concentrations after 48 h of growth did not further induce the already elevated expression levels (Figure 5C).

It is noteworthy that the addition of inhibitory concentrations of different oxidative and nitrosative stress inducing agents as well as limiting the amount of glucose and yeast extract did not increase the expression of MT in the exponential or the stationary growth phase, ruling out the potential role of MT in stress response (data not shown).

### MT Is Not Crucial for Biofilm Formation, Motility, and Viability

In contrast to planktonic cultures, an MT is found among the differentially expressed genes in *P. aeruginosa* biofilms (Hentzer et al., 2005; Waite et al., 2005, 2006). Therefore, we investigated the importance of PflQ2 MT for biofilm formation. Interestingly, *P. fluorescens* Q2-87 cells formed a biofilm in microtiter plates only at 10°C but not at 30°C. No significant difference in biofilm formation between the wild-type Q2-87 and the ΔMT mutant strain was observed, neither under normal growth conditions nor in the presence of inhibitory concentrations of Zn^II^ or Cd^II^ (Supplementary Figure S9). Since biofilm formation is strongly influenced by cell motility, the swarming and swimming abilities of the two strains were tested. No significant difference in swimming was observed between the wild type and mutant in the presence or absence of inhibitory concentrations of Zn^II^/Cd^II^ (Supplementary Figure S10). The strains did not exhibit swarming motility under any of the tested conditions. Taken together, our results suggest that PflQ2 MT is not required for biofilm formation in *P. fluorescens* Q2-87.

As many proteins synthesized at the transition to the stationary phase are involved in survival during nutrient deprivation, the role of MT in the viability of *P. fluorescens* Q2-87 was analyzed by determining the viable cell counts of the wild type and mutant strain. No difference in the number of viable cells was observed after 24 and 48 h incubation (data not shown), suggesting that the MT is not essential for starvation survival of *P. fluorescens* over this time period.

### MT Is Important in the Long-Term Survival of *P. fluorescens*

**FIGURE 6:**
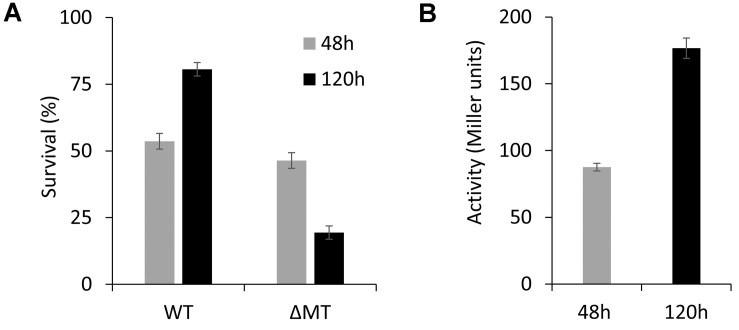
WT vs. ΔMT competition study. **(A)** Percentage of survived bacteria after 48 h (WT:ΔMT = 54:46) and 120 h (WT:ΔMT = 81:19) incubation of cultures containing the mixed WT and ΔMT strains. **(B)** Expression levels of MT under the conditions given in **(A)**.

Our finding that MT expression is upregulated in the stationary growth phase suggests that MT can provide a competitive advantage to *P. fluorescens* in long-term survival. To investigate this, competition studies between a mCherry-marked wild-type derivative and the ΔMT mutant strain were performed in modified ABG media containing 50 μM Zn^II^. The viability of the bacteria was determined after 48 and 120 h by plating on LB medium. Our results show that there is no significant difference in the cell counts after 48 h (Figure 6A). However, we observed that there was a decline in the viability of the ΔMT mutant by ∼20% after 120 h, which can be correlated to the increased expression of PflQ2 at the same time point (Figures 6A,B), suggesting that the MT confers a competitive advantage for the long-term survival of *P. fluorescens*.

## Conclusion

Since most studies on metal ion homeostasis and detoxification in *Pseudomonas* species were performed during the exponential phase of bacterial growth, the role of PsdMTs has remained elusive. Here, we investigated the role of MT in zinc homeostasis and cadmium detoxification using *P. fluorescens* Q2-87 as a model strain. Intriguingly, significant MT expression was detected only after 12 h of incubation when bacterial cultures entered the stationary growth phase. Therefore, the MT does not seem to play a role in zinc homeostasis and cadmium detoxification in *P. fluorescens* during exponential growth. However, our competition studies provide evidence that the MT significantly supports the long-term viability of *P. fluorescens* Q2-87. It is generally assumed that the majority of the bacteria experience nutrient deprivation, exposure to toxic waste products and various stresses such as pH, temperature and osmolality in their natural environments (Kram and Finkel, 2014; Jaishankar and Srivastava, 2017). Consequently, bacteria induce the expression of specific genes that help to increase their competitive fitness. Our finding that exposure to inhibitory concentrations of zinc and cadmium ions increased MT expression in the stationary phase suggests that MT may play a role in the storage or sequestration of metals when present in excess. In conclusion, this study reinforces the importance of MTs in metal homeostasis in *P. fluorescens* and provides evidence for a specific role in starvation survival.

## Data Availability Statement

All datasets presented in this study are included in the article/Supplementary Material.

## Author Contributions

JH planned and performed the experiments and wrote the manuscript. AM planned and supervised the experiments and helped writing the manuscript. LE planned the experiments and helped writing the manuscript. EF planned and discussed the experiments and wrote the manuscript. All authors contributed to the article and approved the submitted version.

## Conflict of Interest

The authors declare that the research was conducted in the absence of any commercial or financial relationships that could be construed as a potential conflict of interest.

## Abbreviations

bacMTs: bacterial metallothioneins
CFU: colony-forming units
DMSO: dimethyl sulfoxide
EDTA: ethylenediaminetetraacetic acid
F-AAS: flame atomic absorption spectrometry
GmR: gentamycin resistance
LB: lysogeny broth
MIC: minimum inhibitory concentration
MT: metallothionein
ONPG: ortho-nitrophenyl- β-galactosidase
PCR: polymerase chain reaction
PflQ2 MT: metallothionein from *Pseudomonas fluorescens* Q2-87
PsdMT: *Pseudomonas* metallothionein
SDS: sodium dodecyl sulfate
TPEN: N,N,N’,N’-tetrakis(2-pyridinylmethyl)-1,2-ethanediamine
TpR: trimethoprim resistant.
