# Supplementary material for: Deciphering the Enigmatic Function of Pseudomonas Metallothioneins
Source: Front Microbiol. 2020 Jul 22;11:1709. doi: 10.3389/fmicb.2020.01709 (PMC7387436; doi:10.3389/fmicb.2020.01709)
Supplement: Supplementary file 1 [file Data_Sheet_1.PDF]

Supplementary Information

to

Deciphering the enigmatic function of *Pseudomonas* metallothioneins

Jelena Habjanič,[1] Anugraha Mathew,[2] Leo Eberl,[2*] Eva Freisinger[1*]

[1] *Department of Chemistry, University of Zurich, Zurich, Switzerland*

[2] *Department of Plant and Microbial Biology, University of Zurich, Zurich, Switzerland*

Email: leberl@botinst.uzh.ch, freisinger@chem.uzh.ch

Table of Content

Table S1. Bacterial strains used in this study S3

Table S2. Plasmids used in this study S3

Table S3. Primers used in this study S4

Figure S1. Agarose gel of PCR product confirming successful deletion of the MT gene S5

Figure S2. Scheme of *P. fluorescence* Q2-87 knock-out strain construction. S6

Figure S3. Probable promotor region of MT operon from *P. fluorescens* Q2-87 .... S6

Figure S4. Minimal inhibitory concentrations of Zn^{II} and Cd^{II} S7

Figure S5. The effect of Zn^{II} and Cd^{II} on *P. fluorescens* growth (Figure 3) S7

Figure S6. Determination of promotor region of MT operon from *P. fluorescens* Q2-87S5 S8

Figure S7. MT expression in different stages of bacterial growth (Figure 4) S9

Figure S8. MT expression in the stationary and exponential growth phase (Figure 5) S10

Figure S9. Biofilm formation in absence of metal ions and in presence of Zn^{II} or Cd^{II} S11

Figure S10. Swimming motility in absence of metal ions and in presence of Zn^{II} or Cd^{II} S11

References S12

Table S1. Bacterial strains used in this study.

Strain	Genotype/Description	Reference
Pseudomonas fluorescens		
Q2-87	Wheat rhizosphere isolate, wild type	[1]
Q2-87 ΔMT	unmarked MT deletion mutant	This study
Q2-87_mCherry		This study
Q2-87 ΔMT_mCherry		This study
Escherichia coli		
SY327 λpir	araD, Δ(lac pro) argE(Am) recA56 rif^{R}nalA	[5]
Top 10		Invitrogen
S17-1	recA pro hsdR, RP4-Tc::Mu-Km::Tn7 integrated into the chromosome	[6]

Table S2. Plasmids used in this study.

Plasmid	Description	Reference
pGPI-SceI	suicide cloning vector designed to introduce a targeted I-SceI restriction site, Tp^R	[2]
pMe6032	shuttle vector, tet^R	[3]
pGPI-SceI_tet	suicide cloning vector designed to introduce a targeted I-SceI restriction site, Tp^R, tet^R	This study
pRK2013	helper plasmid, RK2 derivative, mob$^+$ tra$^+$ori ColE1, Kn^R	[7]
pDAI-SceI	expressing I-SceI nuclease, tet^R	[2]
pSU11	*lacZ* reporter plasmid, Gm^R	[4]
pMRE100	carrying mini Tn7(Gm)$_{Ptac}$-mcherry, Gm^R, Amp^R	[8]
pUX-BF13	Helper plasmid with transposase for integration of the mini-Tn7 element, Amp$_{100}$	[9]

Table S3. Primers used in this study.

Primer	Sequence
Tetracycline resistance cassette	
tet_Pst1_fwd	GGGGCTGCAGTGCTGTAGTGAGTGGGTT
tet_Pst1_rev	GGGGCTGCAGTCGCGTAACTTAGGACTTG
Homology regions that flank MT gene	
MT_up_Kpn1_fwd	GGGGGGTACCGCACATCGAGGAAACACT
MT_up_Xho1_rev	GGGGCTCGAGTCAAGACCCCACCAGTAA
MT_dn_Xho1_fwd	GGGGCTCGAGTTTTCGCTTGAATCCGGG
MT_dn_EcoR1_rev	GGGGGAATTCTTTCTTGCCTGCTTCGGA
Pseudomonas specific primers	
pseudo_16S_fwd	TGCTTGCACCTCTTGAGA
pseudo_16S_rev	CGCCCAGTAATTCCGATT
Construction of *lacZ* fusions	
MT_prom_L_Xho1_fwd	GCGCCTCGAGGTTCGAAGTGAAGGTCGT
MT_prom_S_Xho1_fwd	GCGCAAGCTTCGCCTAAAGAGAACCTCC
MT_prom_HindIII_rev	GCGCAAGCTTGTTCATGCTCGTCCTCCT
lacZ_rev	TGCTGCAAGGCGATTAAG

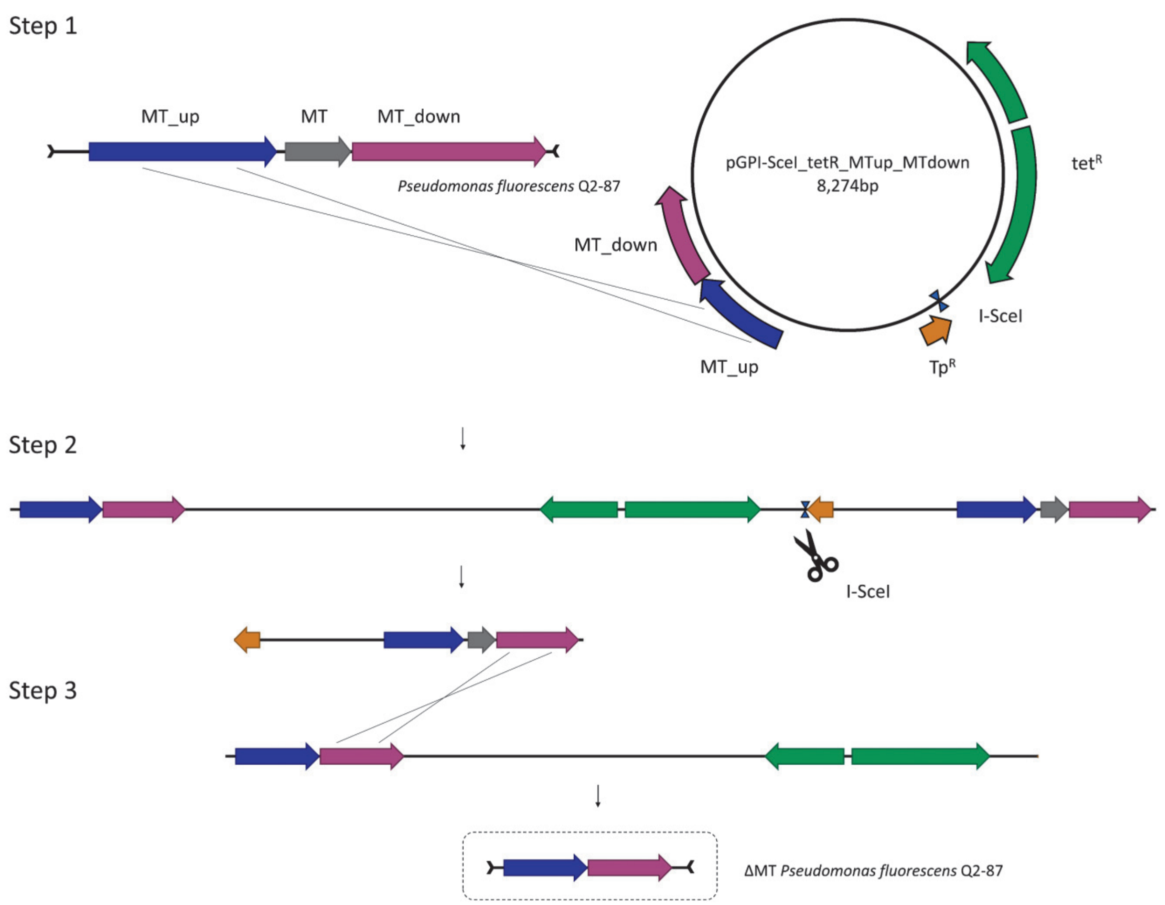

Figure S1. Schematic representation of *P. fluorescens* Q2-87 MT knock-out strain construction.

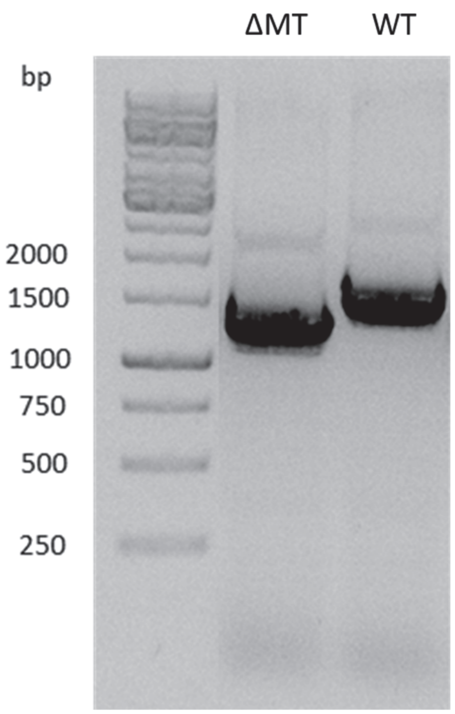

Figure S2. Agarose gel of the PCR product of the MT region amplification in the ΔMT mutant (2nd line) and the wild type (3rd line) confirming successful deletion of the MT gene.

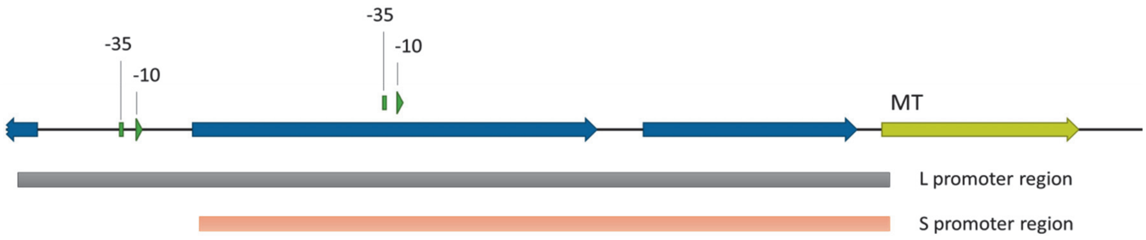

Figure S3. Schematic representation of probable promoter regions of the MT operon in *P. fluorescens* Q2-87.

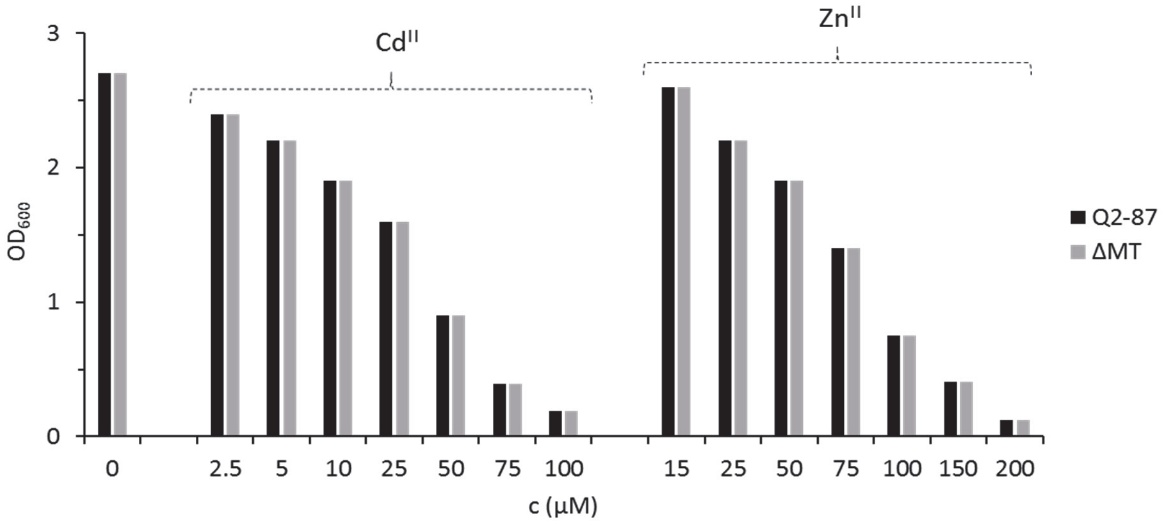

Figure S4. Determination of the minimal inhibitory concentrations (MIC) of zinc and cadmium ions for *P. fluorescens* Q2-87 and its ΔMT mutant after 12 h.

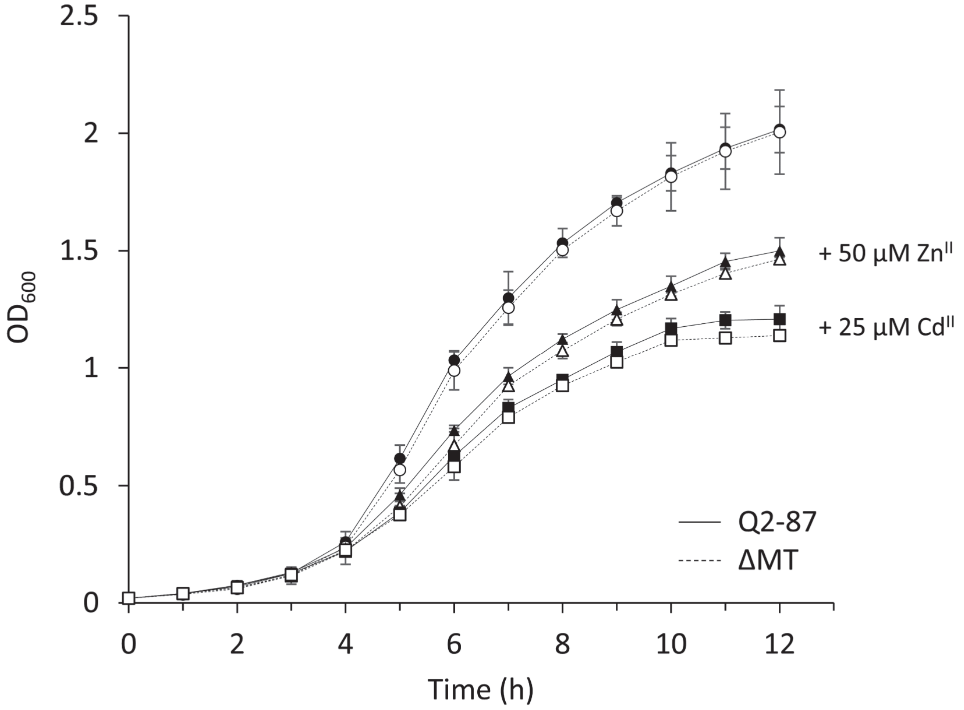

Figure S5. The effect of Zn^{II} and Cd^{II} on *P. fluorescens* growth. Growth of *P. fluorescens* Q2-87 and the ΔMT mutant was compared by optical density measurements at 600 nm upon addition of 50 µM Zn^{II} or 25 µM Cd^{II}. Data are presented as the mean of three independent experiments and the standard deviation is given as error bars. Figure S5 corresponds to Figure 3 in the manuscript, but the y axis is in linear scale.

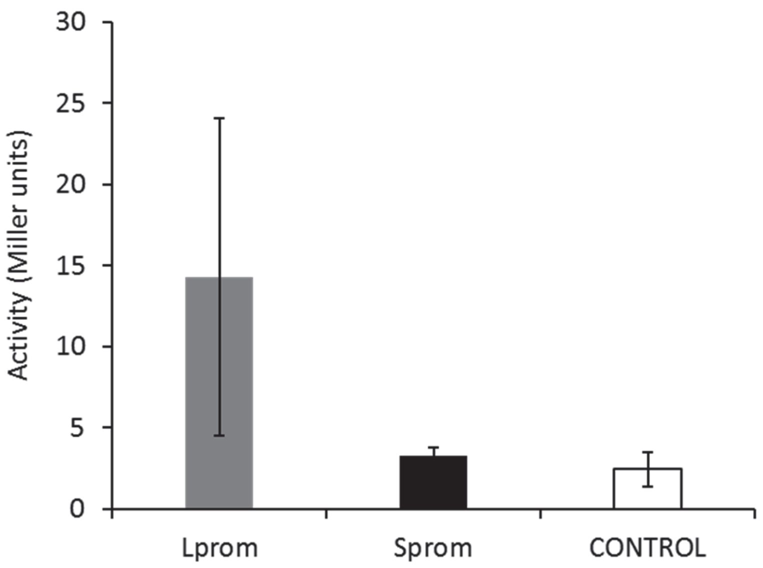

Figure S6. Determination of promotor region for MT operon in *P. fluorescens* Q2-87.

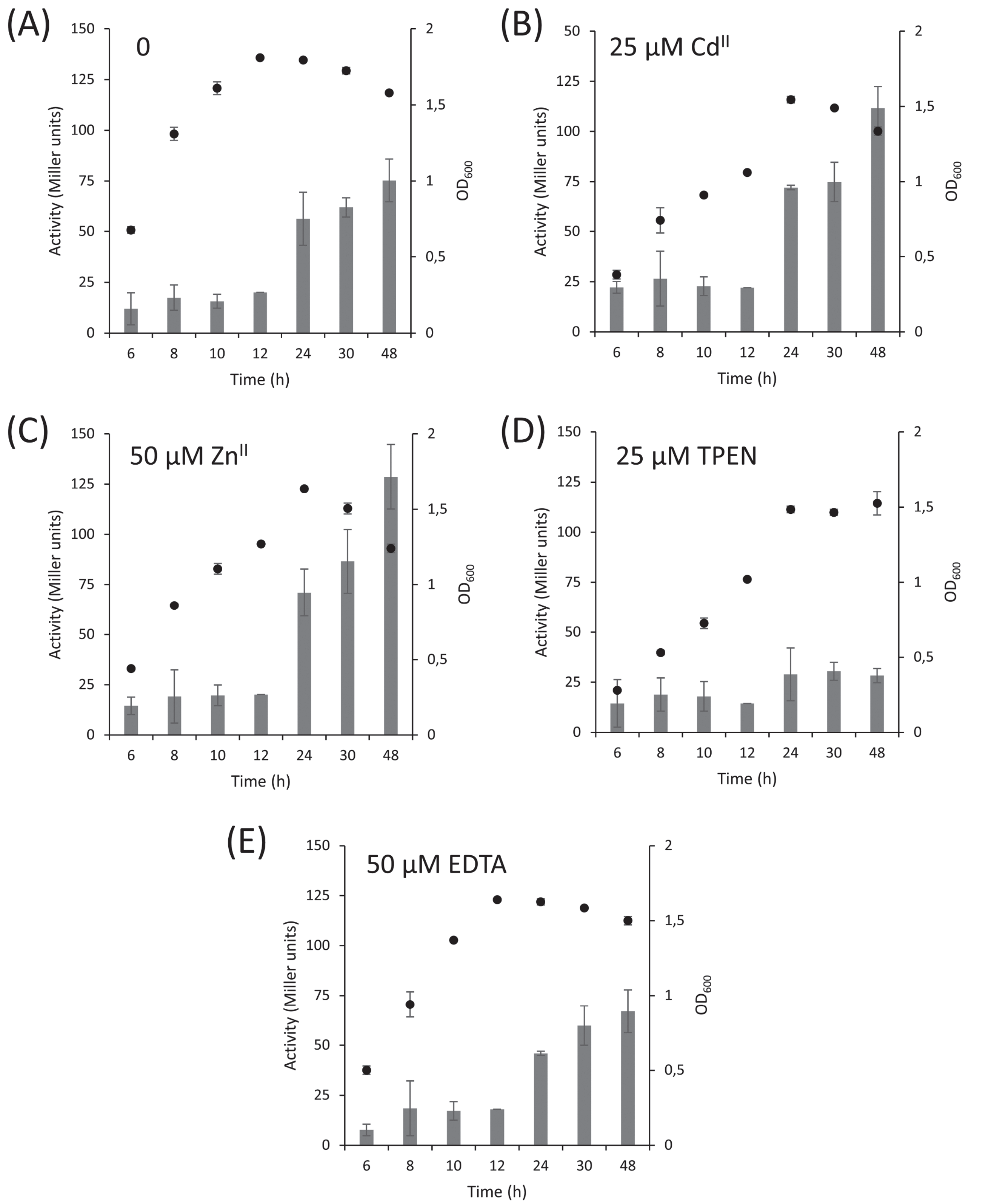

Figure S7. MT expression in different stages of bacterial growth. MT expression in the wild type Q2-87 in modified ABG medium is depicted in form of bars in Miller units with (A) no addition of metals, (B) 25 µM Cd^{II}, (C) 50 µM Zn^{II}, (D) 25 µM TPEN or (E) 50 µM EDTA. The secondary y axis on the right shows the OD_{600} values of bacterial culture at the time samples were taken (black full circles). Figure S7 corresponds to Figure 4 in the manuscript, but the secondary y axes are in linear scale.

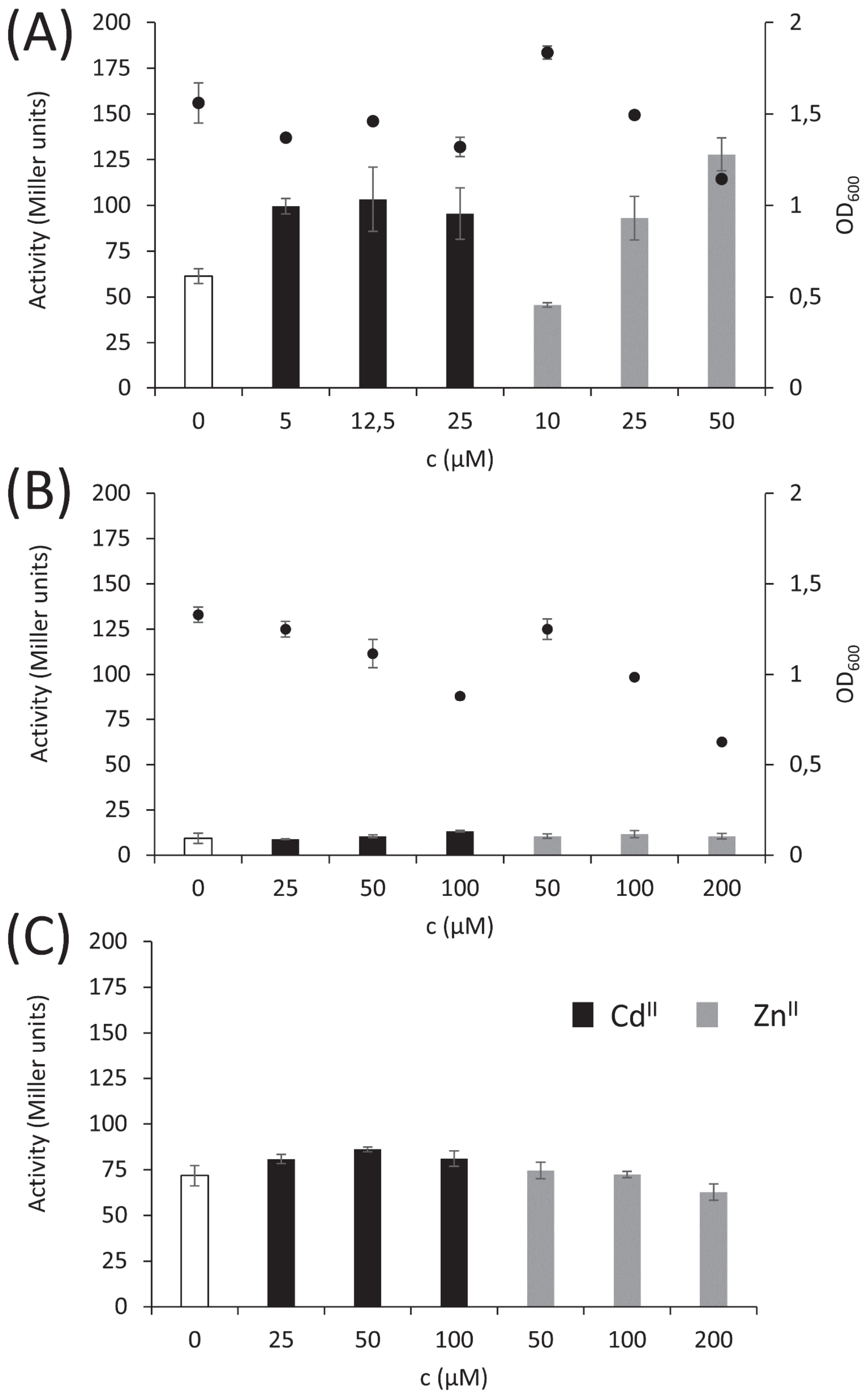

Figure S8. Level of MT expression in the stationary and exponential phase of growth. MT expression without additional metal ions (white bars) or in presence of different concentrations of Zn^{II} (grey bars) or Cd^{II} (black bars) during mid-exponential phase of bacterial growth is given in Miller units and depicted after (A) 48 h incubation or (B) 2 h incubation. In (C) the different concentrations of Zn^{II} or Cd^{II} were added after 48 h of bacterial growth followed by 2 h incubation. The secondary y axis on the right in (A) and (B) represents OD_{600} values at the time samples were taken (black full circles). Figure S8 corresponds to Figure 5 in the manuscript, but the secondary y axes are in linear scale.

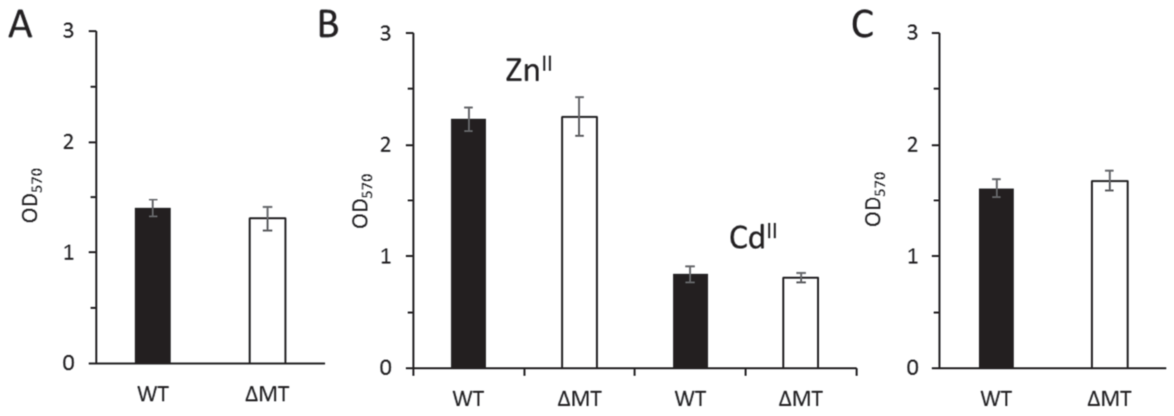

Figure S9. Biofilm formation of Q2-87 (WT; black bars) and ΔMT (white bars) strains at 10 °C for 48 h with A) no additional metal ions added, B) 80 µM Zn^{II} and 30 µM Cd^{II} ions added, C) using bacterial cultures preadapted with 80 µM Zn^{II} ions.

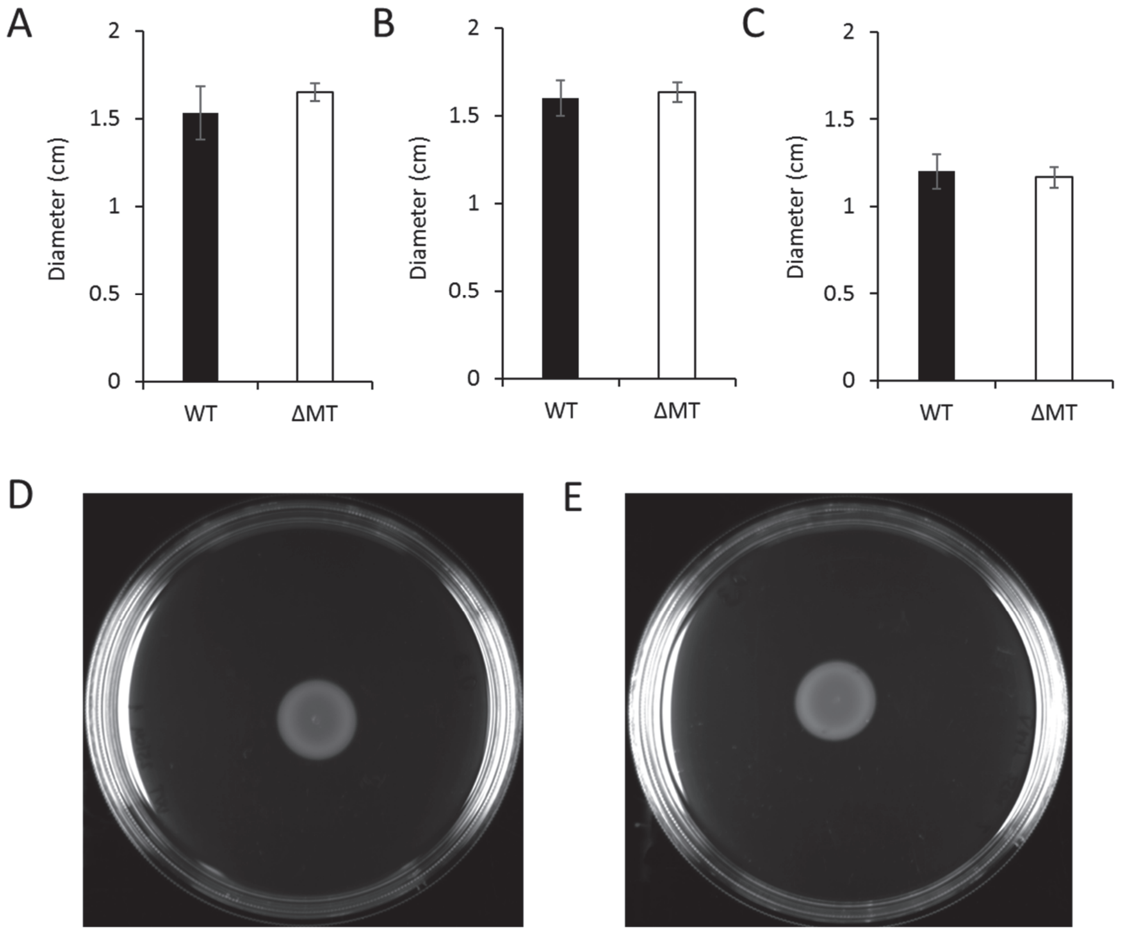

Figure S10. Swimming motility of Q2-87 (WT; black bars) and ΔMT (white bars). A) No additional metal ions added, B) 80 µM Zn^{II} ions added C), 20 µM Cd^{II} ions added. Swimming motility zones of Q2-87 (D) and ΔMT (E) on an agar plate with no additional metal ions added.

References

[1] Loper, J. E.; Hassan, K. A.; Mavrodi, D. V.; Davis, E. W., 2nd; Lim, C. K.; Shaffer, B. T.; Elbourne, L. D.; Stockwell, V. O.; Hartney, S. L.; Breakwell, K.; Henkels, M. D.; Tetu, S. G.; Rangel, L. I.; Kidarsa, T. A.; Wilson, N. L.; van de Mortel, J. E.; Song, C.; Blumhagen, R.; Radune, D.; Hostetler, J. B.; Brinkac, L. M.; Durkin, A. S.; Kluepfel, D. A.; Wechter, W. P.; Anderson, A. J.; Kim, Y. C.; Pierson, L. S., 3rd; Pierson, E. A.; Lindow, S. E.; Kobayashi, D. Y.; Raaijmakers, J. M.; Weller, D. M.; Thomashow, L. S.; Allen, A. E.; Paulsen, I. T., *PLoS Genet.*, 2012, **8**, e1002784.
[2] Flannagan, R. S.; Linn, T.; Valvano, M. A., *Environ. Microbiol.*, 2008, **10**, 1652-1660.
[3] Heeb, S.; Blumer, C.; Haas, D., *J. Bacteriol.*, 2002, **184**, 1046-1056.
[4] Malott, R. J.; O'Grady, E. P.; Toller, J.; Inhulsen, S.; Eberl, L.; Sokol, P. A., *J. Bacteriol.*, 2009, **191**, 2447-2460.
[5] Miller, V. L.; Mekalanos, J. J., *J. Bacteriol.*, 1988, **170**, 2575-2583.
[6] Simon, R.; Priefer, U.; Pühler, A., *Bio/Technology*, 1983, **1**, 784-791.
[7] Figurski, D. H.; Helinski, D. R., *Proc. Natl. Acad. Sci. USA*, 1979, **76**, 1648-1652.
[8] Remus-Emsermann, M. N.; Gisler, P.; Drissner, D., *FEMS Microbiol. Lett.*, 2016, **363**, fnw178.
[9] Bao, Y.; Lies, D. P.; Fu, H.; Roberts, G. P., *Gene*, 1991, **109**, 167-168.
